# Multiple subungual glomus tumors

**DOI:** 10.1016/j.jdcr.2026.04.060

**Published:** 2026-05-06

**Authors:** Hsin Jen Faith Chow, Robert Love, Minh Lam, Jamie Von Nida

**Affiliations:** a Department of Dermatology, Sir Charles Gairdner Hospital, Nedlands, Australia; b Department of Plastic Surgery, Sir Charles Gairdner Hospital, Nedlands, Australia; c Cutaneous Pathology, Perth, Australia

**Keywords:** digital, fingernail, glomus tumor, multiple, nail, neurofibromatosis type 1, subungual, thumbnail, toenail

## Introduction

Subungual glomus tumors are rare, benign vascular neoplasms that typically present as solitary lesions.^1^ Multiple glomus tumors are uncommon and, when present, are more frequently extradigital or associated with neurofibromatosis type 1 (NF1).^2^ Although an association between NF1 and multiple glomus tumors is recognized, reports of multiple subungual glomus tumors remain scarce, particularly in patients without clinical features of NF1. We report a case of metachronous subungual glomus tumors affecting both a fingernail and a toenail in an NF1-negative patient and review the existing literature. This case highlights the diagnostic challenge of subungual glomus tumors and underscores the need for heightened clinical suspicion in patients with chronic nail pain.

## Case report

**Fig 1:**
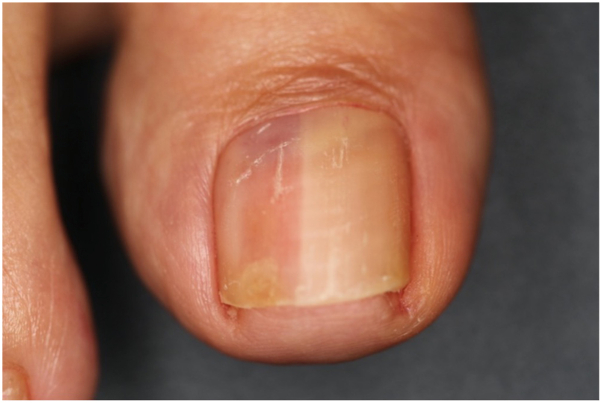
Right great toenail with erythronychia and proximal violaceous discoloration.

A 35-year-old woman presented with 3 years of sharp, paroxysmal, localized pain involving the right great toenail. Symptoms were exacerbated by pressure and cold exposure. Clinical examination demonstrated a positive Hildreth’s and Love’s pin test, thinning of the nail plate, and a well-demarcated longitudinal erythronychia with proximal violaceous discoloration (see Fig 1). There were no clinical features of NF1, no personal or family history of NF1 or glomus tumors; a formal NF1 genetic study was not performed.

**Fig 2:**
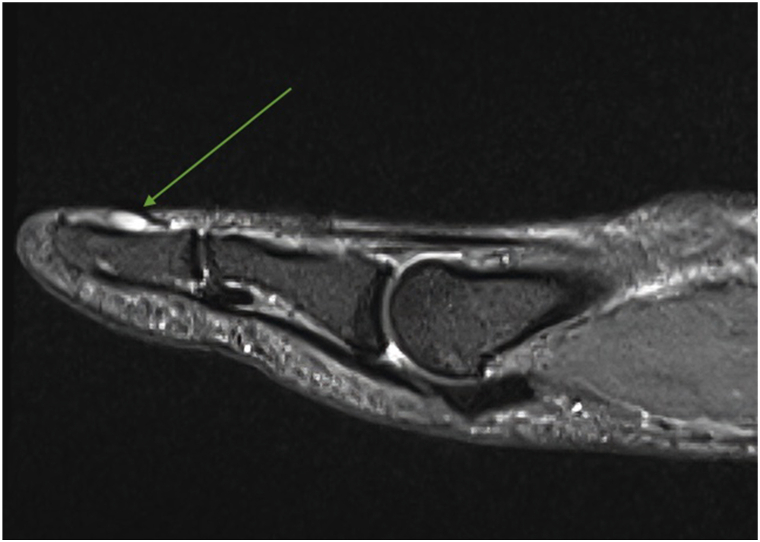
Magnetic resonance imaging of the right great toenail showcasing a glomus tumor as indicated by the *arrow*.

Magnetic resonance imaging demonstrated a 7 mm × 5 mm × 2 mm well-circumscribed nodule deep to and within the nail bed at the distal phalanx, consistent with a glomus tumor (see Fig 2). The lesion was excised en bloc, including the thinned, involved nail bed and reconstructed with a split-thickness nail bed graft from the adjacent area on the same digit. Histopathology demonstrated a well-circumscribed tumor composed of uniform, round to polygonal glomus cells arranged in solid sheets and nests. The cells exhibited centrally placed, round nuclei with inconspicuous nucleoli and amphophilic cytoplasm. The glomus cells were concentrically distributed around vascular channels, producing a characteristic perivascular arrangement. The vascular endothelium was unremarkable, and the intervening stroma showed mucinous change. No mitotic figures were identified, and immunohistochemical staining revealed diffuse tumor cell positivity for smooth muscle actin. These findings confirmed the diagnosis of glomus tumor.

**Fig 3:**
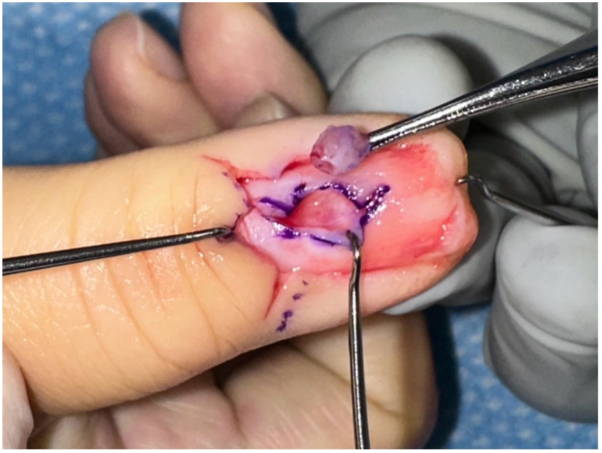
Surgical excision of the right thumbnail glomus tumor.

**Fig 4:**
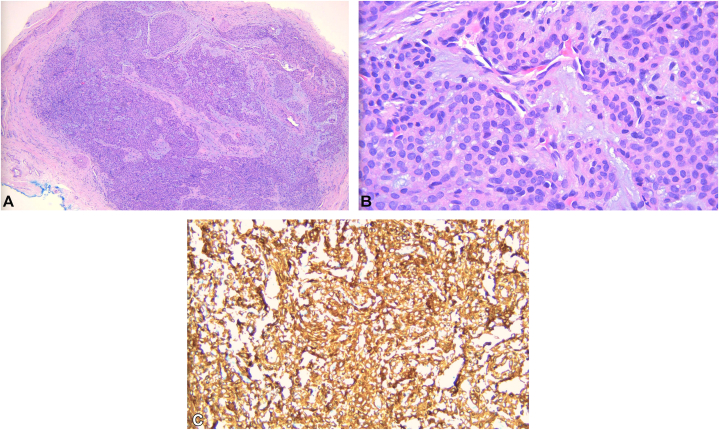
Histopathologic features of the right thumb glomus tumor. **A,** Histologic section of the right thumb tumor demonstrates a well-circumscribed tumor composed of uniform cells arranged in solid sheets and nests (hematoxylin and eosin stain; original magnification×50). **B,** Higher magnification shows glomus cells with regular round nuclei, indistinct cytoplasmic borders, and amphophilic cytoplasm, arranged in nests and in a perivascular pattern (hematoxylin and eosin stain; original magnification×400). **C,** Immunohistochemical staining of the right thumb lesion reveals diffuse positivity for smooth muscle actin in tumor cells.

Thirteen months after initial presentation, the patient developed similar symptoms involving the right thumbnail, including focal pain and cold hypersensitivity. Examination revealed subtle violaceous discoloration at the proximal aspect of the thumbnail, with a positive Love’s pin test. Magnetic resonance imaging demonstrated a 4 mm × 3 mm × 2 mm lesion within the nail bed, consistent with a second glomus tumor. Surgical removal via zigzag nail bed incision was performed, allowing direct closure and preserving nail plate adherence (see Fig 3). Histopathology and immunohistochemical staining (see Fig 4) were the same as the right great toe lesion, confirming glomus tumor.

At 5 months following the second excision, the patient remained symptom-free with no evidence of recurrence at either site, with slight dystrophic regrowth of the toenail. However, given the relatively short follow-up duration, longer-term surveillance is required to more definitively assess for recurrence and overall outcome.

## Discussion

Glomus tumors are rare, generally benign tumors which arise from the glomus body located in the deep dermis and can occur as solitary or multiple lesions.^1^ The classic triad of symptoms includes severe paroxysmal pain, pinpoint tenderness, and cold hypersensitivity,^1^ all of which were present in our patient. Other special tests on examination that aid diagnosis are cold sensitivity test, Hildreth’s test, and Love’s pin test. Further investigations include magnetic resonance imaging, ultrasound and x-ray.^1^ Management for subungual glomus tumors is surgical excision, which usually resolves symptoms and recurrence, typically due to incomplete excisions.^1^

Solitary glomus tumors most commonly affect digits,^1^, ^2^, ^3^ whereas patients with multiple glomus tumors usually experience these in extradigital regions such as the lower limbs, or proximal upper limbs^1^ or a combination of digital and extradigital sites.^1^^,^^2^ Purely multiple subungual glomus tumors are rarely reported. Review of literature to date identified 8 cases of histologically confirmed multiple subungual glomus tumors, summarized in Table I.^2^, ^3^, ^4^, ^5^, ^6^, ^7^, ^8^, ^9^ Notably, this case appears to be the only described instance involving a toenail and a fingernail. Most reported patients, including the present case, experienced pain for several years before diagnosis, highlighting the diagnostic delay associated with this condition.

**Table I:** Summary of clinical features and outcome of multiple subungual glomus tumors

Reference	Age (y)	Sex	No. of glomus tumors	Glomus tumor location	Pain	Cold hypersensitivity	Duration of symptoms	NF1 association	Outcome
Di Chiacchio et al,^4^ 2012	32	M	2	Left middle finger	Y	Y	18 mo	N/NR	Symptoms resolved, no recurrence at 2 mo
Ferrari et al,^2^ 2023	56	F	7	7 unspecified fingers	Y	Y	8 y	Y	Recurrence in 1 nail, re-excision performed Symptoms resolved, no recurrence 6 mo post re-excision
Ghosh et al,^3^ 2011	63	F	2	Left ring and little finger	Y	Y	6 y	Y	Symptoms resolved, no recurrence (FU NR)
Graham and Wolff,^5^ 1992	64	F	2	Right middle and ring finger	Y	Y	Several years	N/NR	Symptoms resolved, no recurrence at 3 mo
Lee et al,^6^ 2017	34	M	5	Right middle and ring finger. Left thumb, middle, ring, and little finger	Y	NR	5 y	N/NR	Symptoms resolved, no recurrence at 1 y
Morohashi et al,^7^ 2015	45	F	3	Left ring finger and thumb. Right ring finger	Y	Y	6-12 mo	Y	Symptoms resolved, no recurrence (FU NR)
Okada et al,^8^ 1999	22	F	5	Left index, middle, ring, and little finger. Right ring finger	Y	Y	3 y	Y	Symptoms resolved, no recurrence (FU NR)
Yuen et al,^9^ 2015	43	M	3	Left ring and little finger. Right little finger	Y	Y	5 y	Y	NR

*F*, Female; *FU*, follow-up; *M*, male; *N*, nil; *NF1*, neurofibromatosis type 1; *NR*, not reported; *Y*, yes.

As time has gone on, increasing reports of glomus tumors and NF1 have prompted further study into the molecular genetic association between the 2.^2^ Patients with NF1 often develop multiple tumors (affecting both digital and extradigital sites) rather than solitary, consistent with an NF1-driven predisposition to a multiple tumor phenotype.^2^^,^^3^ These glomus tumors are caused by biallelic inactivation in α-actin-positive smooth muscle cells of the glomus body.^2^ Thus, in patients with NF1 presenting with painful subungual lesions, clinicians should maintain a high index of suspicion for glomus tumors; conversely, the presence of multiple digital glomus tumors should prompt evaluation for features of NF1.

Of the 8 reported cases of multiple subungual glomus tumors, 5 were associated with NF1, while 3 did not report, or had no clinical or genetic evidence of NF1 (as shown in Table I). While subungual glomus tumors are classically solitary lesions and multiple glomus tumors typically described in extradigital regions or NF1-associated patients, it could be possible that NF1-negative individuals with multiple subungual tumors might represent a distinct subgroup; however, this remains speculative and requires further evidence. Agaram et al^10^ explored gene rearrangements associated with glomus tumors, namely NOTCH gene mutations. NOTCH gene fusions were identified in over half of cases; however, they were absent in subungual tumors.^10^ Of the subungual cases, Agaram et al^10^ did not specify whether these were solitary or multifocal lesions, nor whether they were associated with NF1. Further molecular analysis is therefore required to explore any potential differences in NF1-negative multiple subungual glomus tumors.

Multiple subungual glomus tumors are exceptionally rare, particularly in the absence of NF1. This case underscores the importance of considering glomus tumors in patients with chronic subungual pain and of evaluating for NF1 when multiple lesions are present.

## Conflicts of interest

None disclosed.
